# A Critical Balance: dNTPs and the Maintenance of Genome Stability

**DOI:** 10.3390/genes8020057

**Published:** 2017-01-31

**Authors:** Chen-Chun Pai, Stephen E. Kearsey

**Affiliations:** 1 CRUK-MRC Oxford Institute for Radiation Oncology, Department of Oncology, University of Oxford, ORCRB, Roosevelt Drive, Oxford OX3 7DQ, UK; chen-chun.pai@oncology.ox.ac.uk; 2 Department of Zoology, University of Oxford, South Parks Road, Oxford OX1 3PS, UK

**Keywords:** dNTP, DNA polymerase, genome instability, ribonucleotide reductase, replication fidelity, proofreading

## Abstract

A crucial factor in maintaining genome stability is establishing deoxynucleoside triphosphate (dNTP) levels within a range that is optimal for chromosomal replication. Since DNA replication is relevant to a wide range of other chromosomal activities, these may all be directly or indirectly affected when dNTP concentrations deviate from a physiologically normal range. The importance of understanding these consequences is relevant to genetic disorders that disturb dNTP levels, and strategies that inhibit dNTP synthesis in cancer chemotherapy and for treatment of other disorders. We review here how abnormal dNTP levels affect DNA replication and discuss the consequences for genome stability.

## 1. Introduction

**Figure 1:**
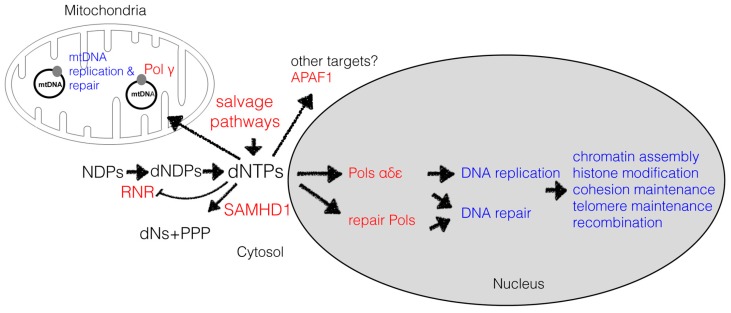
Overview of dNTP levels and impact on cellular processes. dNTP levels depend on a balance between synthesis, consumption, and degradation. Consumption of dNTPs in DNA synthesis influences a wide range of activities due to the relationship between DNA replication and other chromosomal processes. As well as affecting DNA polymerase function, dNTPs may target other proteins such as APAF1. (APAF1: Apoptotic protease activating factor 1; dNs: deoxyribonucleosides; PPP: triphosphate; RNR: ribonucleotide reductase).

Defects in genome maintenance, recognised as an enabling characteristic in cancer, contribute to the development of some neurodegenerative conditions and may be a significant factor in normal ageing. Genome instability may result from a wide range of defects affecting DNA replication, repair, checkpoint pathway function, and chromosome segregation (summarised in recent reviews [1,2,3,4,5,6,7,8,9]) and this review focuses on recent developments regarding one specific aspect, namely how abnormal levels of dNTPs may compromise genome stability. A high dNTP concentration has long been recognised as a factor reducing the fidelity of DNA polymerase proofreading, but recently it has become more widely appreciated that cellular disturbances in dNTPs may affect genome integrity in diverse ways [10]. This reflects the fact that DNA replication impinges on many chromosomal activities, such as DNA repair, recombination, and chromatin assembly (Figure 1) and thus derangements in dNTP levels may impact on a wide range of processes. This review will also summarise the clinical and physiological situations which may lead to derangement of dNTP levels in human cells (a broad survey of this area is provided in [10,11,12]).

## 2. Overview of dNTP Levels and DNA Synthesis

Initiation of DNA replication in eukaryotes involves the assembly of pre-replicative complexes (pre-RCs) on chromatin in late mitosis/G1, followed by initiation of DNA synthesis in S phase ([13], reviewed in [14]). Pre-RC formation requires the origin recognition complex (ORC), a heterohexameric complex of proteins which in *Saccharomyces cerevisiae* binds to DNA in a sequence-specific manner, but in other eukaryotes shows little or no sequence specificity [15]. Two Mcm2-7 hexamers are assembled at ORC in a step involving Cdc6, which binds to the ORC complex on chromatin, and Cdt1, which binds to Mcm2-7 and opens the hexameric complex to facilitate assembly onto DNA. Following Mcm2-7 assembly, both Cdt1 and Cdc6 are displaced in a step requiring ORC-mediated ATPase activity. Subsequently, cyclin-dependent kinase (CDK) activation allows the association of Cdc45, GINS, and DNA polymerase ε (Pol ε) with pre-RCs, and activation of the Mcm2-7-GINS-Cdc45 helicase requires Mcm2-7 phosphorylation by Cdc7-Dbf4 (DDK) kinase. DNA unwinding allows priming by Pol α followed by elongation, most probably by Pol ε on the leading strand and Pol δ on the lagging strand [16,17], although a recent controversial paper suggests that Pol δ may execute both leading and lagging strand synthesis [18].

Activating the enzymes involved in DNA unwinding and DNA synthesis must be coordinated with upregulation of the dNTP supply, as the dNTP pool in S phase is only sufficient for replicating a small fraction of the genome [19,20]. This is achieved in part by upregulation of ribonucleotide reductase (RNR) activity which occurs by various mechanisms, including allosteric activation, increased levels of RNR expression, altered cellular localisation of RNR subunits, and proteolysis of RNR inhibitory proteins (reviewed in [21,22,23,24]). Nucleotide salvage pathways also contribute to dNTP replenishment and these are particularly important for neuronal cells [25]. In mammalian cells, an additional factor regulating dNTP levels is SAMHD1 (SAM And HD Domain Containing Deoxynucleoside Triphosphate Triphosphohydrolase 1), a dNTP hydrolase that maintains low levels of dNTPs outside of S phase, but which is proteolysed in S phase ([26], reviewed in [27]). Maintaining dNTP concentrations at levels optimum for replicative fidelity may also be assisted by temporal regulation of initiation during S phase. Not all potential replication origins are used in S phase, and activation of origins is temporally regulated, so that some initiate early and others late, thus limiting the number of replication forks that are active at any one time and moderating the demand for dNTPs (reviewed in [28]). The synthesis of deoxynucleotides in the cytoplasm is also important for mitochondrial DNA (mtDNA) synthesis, and import of nucleosides/nucleotides via several mitochondrial transporters, together with mitochondrial salvage pathways, provide a separate pool of dNTPs for mtDNA replication and repair [10].

## 3. Effects of High dNTP Levels on Cell Cycle Progression, DNA Replication and Repair

High in vivo levels of dNTPs can be experimentally induced by inactivating dATP feedback inhibition of RNR [29,30], deleting small protein RNR inhibitors [31,32], over-expressing RNR subunits [33], or by inactivating mammalian SAMHD1 [34]. In addition, DNA damage induces upregulation of dNTP levels in bacteria [35] and also in yeast [30,36,37], although mammalian cells show little change in dNTP levels on DNA damage induction [38]. High dNTP levels are detrimental to the fidelity of DNA replication in bacteria [35], yeast [30,39] and mammalian cells [40,41]. This reflects, at least in part, the propensity of DNA polymerases to extend a mismatched primer-template and reduced efficiency of proofreading at high nucleotide levels [42,43]. In vivo, an additional factor appears to be stimulation of DNA synthesis by inaccurate translesion synthesis (TLS) polymerases (reviewed in [44]). The ability of TLS polymerases to take over from normal replicative polymerases may be facilitated by high dNTP levels, since they have a higher K_m_ for dNTPs than Pol δ and Pol ε [45,46] and accordingly, inactivating TLS polymerases reduces the mutation rate associated with increased dNTP levels [35,39]. Consistent with increased efficiency of replication on damaged templates, increased dNTP levels in yeast leads to improved resistance to DNA damage caused by UV and 4NQO [30,39] which is primarily repaired by nucleotide excision repair (NER). Confusingly, in *Schizosaccharomyces pombe*, high dNTP levels lead to increased sensitivity to DNA damage caused by camptothecin and MMS [39]. Here, repair involves DNA synthesis in homologous recombination, but it is not clear why this pathway should be adversely affected by high dNTP levels [47].

Thus, at least in yeasts, defects in DNA replication and repair factors can trigger increased dNTP levels, and the consequent increased mutation rate may arise from a combined effect of the primary replication/repair defect as well as the change in dNTP levels [37,48,49]. A “vicious-circle” scenario is seen with error-prone Pol δ and Pol ε mutants which trigger increases in dNTP pools via activation of the S-phase checkpoint, and the mutation rate of the variant polymerases is subsequently further enhanced by the higher dNTP levels [48,50].

Increasing dNTP levels also decreases the length of S phase under unstressed conditions, implying that physiological nucleotide levels are limiting for DNA synthesis, a finding which is consistent with analysis of DNA synthesis rates in vitro [51,52]. Direct analysis of fork rate in *S. cerevisiae* shows that elevating dNTP levels facilitates replication of damaged templates and may prevent activation of the DNA damage checkpoint pathway [47]. It is not clear why S phase is longer than the minimum necessary time (see [28]), but moderating the rate of DNA synthesis by limiting dNTP levels not only provides a higher fidelity of synthesis [52] but may also facilitate other aspects of replisome function, such as facilitating the propagation of epigenetic histone modifications in S phase (see below).

In addition to affecting the rate and fidelity of S phase, high dNTP levels can delay entry into S phase. In *S. cerevisiae*, very high levels of dNTPs resulting from overexpression of an RNR variant not subject to dATP inhibition (D57N mutation in the large subunit) causes a delay to S phase entry that appears to act before the Cdc45 loading step at initiation [33]. The mechanism of this delay is unclear but does not involve activation of DNA damage or replication checkpoints. High RNR activity may also lead to increased dUTP incorporation into DNA which is potentially mutagenic [53,54]. This occurs as overproduction of dUDP by RNR may overwhelm the pathway converting this nucleotide to dTTP, with dUDP conversion to dUTP instead, allowing incorporation into DNA. In mammalian cells, dNTP levels are downregulated outside of S phase by activation of the SAMHD1 dNTP hydrolase. Inactivation of this enzyme also prevents S phase entry, presumably due to elevated dNTP levels, but again the mechanistic link between high dNTPs and the block to DNA synthesis is obscure [34]. It is not clear why dNTP levels are reduced outside of S phase. Plausibly this is a strategy to prevent viral replication in nonproliferating cells, since a reduced SAMHD1 level causes increased susceptibility to lentiviral infection [26,55].

## 4. Effects of Low dNTP Levels on DNA Replication and Repair

Depletion of dNTPs, effected by hydroxyurea (HU)-mediated RNR inhibition for instance, results in a global inhibition of DNA replication and fork stalling. Arrest of DNA synthesis may occur before exhaustion of nucleotide pools, [56], and this could serve to preserve dNTPs for DNA damage repair, or alternatively prevent DNA synthesis under suboptimal conditions. Alternatively, this could simply reflect the fact that global dNTP measurements may not give a good indication of nucleotide levels available to polymerases due to cell-to-cell or subcellular variations in concentration. DNA helicase is also paused by polymerase stalling [57], and ssDNA exposed at the fork [58] leads to checkpoint activation (reviewed in [59,60]). Checkpoint activation may in part compensate for dNTP starvation by upregulating the nucleotide supply [37,48], reducing initiation at late origins in *S. cerevisiae* [61], and slowing the rate of elongation [62]. In mammalian cells, the origin response to lowering of dNTP levels appears to be distinct from yeast, in that normally dormant origins are activated, with an overall increase in the density of initiation events [63]. Arrested forks appear to be quite stable and replication can resume when dNTP levels are restored, although checkpoint activation may itself lead to genome instability as over-compensated high levels of dNTPs are mutagenic [37]. However, under some circumstances, replisome components may be destabilised and recombination-mediated mechanisms are thought to lead to fork restart. Such mechanisms have been extensively reviewed and are not discussed here [64,65,66,67].

Paradoxically, checkpoint pathway activation has been reported to lead to downregulation of dNTP availability in mammalian cells [68]. Downregulation of Chk1 leads to Mus81/Eme2/Mre11-dependent DNA damage which, via activation of the ATM pathway, appears to limit dNTPs available for replication and results in slower replication fork progression. Curiously, this seems to depend on upregulation or changed subcellular localization of the p53R2 RNR subunit (see below) but the details of this link are not clear.

Intermediate levels of dNTP starvation, while not imposing a global block to DNA synthesis, can have a more pronounced effect on specific genomic regions such as hard-to-replicate sequences, fragile sites, and regions of low sequence complexity [69,70]. For example, cells deficient in Pif1 helicase are sensitive to low dNTP conditions, possibly as Pif1 plays a role in unwinding G-quadruplex (G4) motifs under these conditions [71]. Under low dNTP conditions, the 5’-3’ Chl1/DDX11 helicase appears to be required to maintain fork progression, as does Ctf4, which plays a role in addition to its function in recruiting Chl1 to the replication fork [72,73]. Under-replicated and intertwined DNA regions may persist to mitosis and eventually lead to formation of anaphase bridges or to uneven chromosome segregation [8]. A low level of dNTP also impacts on telomere length homeostasis, with levels of dGTP positively correlating with telomere length [74]. dNTPs may act as prosurvival factors independently of any effect on DNA polymerases. dNTPs inhibit apoptosome formation via an effect on APAF1, thus preventing apoptosis [75].

DNA replication under suboptimal dNTP levels may lead to increased incorporation of ribonucleoside monophosphates (rNMPs). Under normal conditions, rates of misincorporation of rNMPs by Pol α, δ, and ε in vitro are surprisingly high (one in 625, 5000, and 1250, respectively [76]) in spite of efficient active site discrimination [77], and this is likely to be exacerbated by low dNTP levels. rNMP incorporation is the most frequent replication lesion during DNA replication, with more than 1 million rNMPs incorporated during the replication of the mouse genome [78]. rNTPs can slow the replication fork rate via competition with dNTPs, and are likely to increase the rate of mutagenesis as a result [51,79]. Incorporated rNMPs are less well edited by proofreading than incorrect bases [80] and are mainly removed post-replicatively by RNase H2-dependent repair [81,82]. However, an error-prone pathway of rNMP removal by topoisomerase I can lead to small deletions [83,84] and, if rNMPs persist in the template, they cause problems in subsequent DNA synthesis as DNA polymerases tend to stall at such sites [79,85,86].

Propagation of histone modifications may also be affected by dNTP deficiency. Chromatin replication is not just a matter of duplicating DNA sequences, but specific patterns of epigenetic marks on histones affecting gene expression are maintained during the cell cycle [87]. Although mechanisms maintaining histone marks are poorly understood, nucleosomes displaced during DNA replication are thought to be disrupted into (H3-H4)_2_ tetramers and H2A-H2B dimers, and randomly deposited after passage of the fork onto the daughter strands, thus retaining the original histone modifications after replication, albeit diluted [88,89] (reviewed in [90]). However, low dNTPs will result in a slowing of DNA synthesis and potentially extend the period between nucleosome displacement ahead of the helicase and reloading of nucleosomes behind the replisome. This interference with histone recycling may lead to loss of epigenetic inheritance and aberrant gene expression (reviewed in [91]). Evidence for this comes from an assay in which replication fork progression is challenged by a G4 motif under conditions where dNTP levels are reduced by HU. This led to the loss of chromatin marks normally associated with active expression such as H3K4me3, and consequent repression of the reporter gene [92]. Epigenetic instability may also occur by a distinct mechanism, where stalling of replication forks directly induces repressive histone marks during repair of collapsed forks [93,94].

Understanding the consequences of low dNTP levels is important not least as RNR inhibiting drugs such as HU and gemcitabine are used as anti-proliferative agents for treating chronic myeloid leukemia, as well as cervical, bladder, ovarian and breast cancers (reviewed in [95]). The mode of action of these drugs can be rationalised as being selectively toxic to cells having DNA repair or checkpoint deficiencies, and they may also exacerbate oncogene-induced stress due to reduced dNTP levels. There is probably considerable scope for improving the targeting of these therapies by identifying tumours that are sensitive to dNTP starvation. Chronic use of HU in treatment of sickle cell disease, where it stimulates fetal haemoglobin synthesis, allows assessment of the long-term effects of use of an RNR inhibitor [96,97]. HU does not increase the rate of cancer, or the mutation rate of the *HPRT* gene, but a small increase in illegitimate T-cell VDJ-joining events has been detected. These studies, however, did not analyse the effect on dNTP levels in vivo. In cell culture, HU has been reported to increase copy number variants [98] likely to be caused by to fork stalling and subsequent break-induced replication.

## 5. Consequences of Imbalanced dNTP Levels

Not only are the overall levels of dNTPs important for genome stability, but also the balance between individual dNTPs since distortions in dNTP ratios can lead to polymerase incorporation errors [99]. Imbalanced dNTP pools seen in yeast cells expressing different mutant versions of RNR can increase mutation frequencies but do not impede cell cycle progression and can escape detection by the S-phase checkpoint [100,101]. Mismatch repair (MMR) may be saturated by a high rate of misincorporation errors [102], but it is also possible that the accuracy of MMR is affected by imbalanced dNTP pools. Imbalanced dNTP pools cause similar mutation rates on both leading and lagging strands, and mutation rates are higher in coding and late-replicating regions for reasons that are not clear [103]. In mammalian cells, an excess of cellular pyrimidine pool (dCTP) decreases PARP-1 activity and impairs Chk1 activation, leading to under-replicated DNA and ultrafine anaphase bridge formation [104,105]. In contrast, imbalanced dNTP pools caused by depletion of dCTP and dTTP lead to ATR-dependent p53 activation via MMR proteins [106] in advance of any direct effect of replication fork progression. Moreover, incorrect cell cycle regulation of enzymes involved in dNTP synthesis can also lead to deleterious nucleotide imbalances. Thymidine kinase I and thymidylate kinase are involved in dTTP synthesis, and both enzymes are normally degraded by APC/C from mitosis to G1 [107]. Interference with this degradation leads to growth retardation, probably via dTTP-mediated dCTP depletion, abnormally high dTTP and dGTP levels, and an increased mutation rate.

## 6. Clinical and Physiological Aspects of Aberrant dNTP Levels

Aberrant levels of dNTPs can be caused by mutations that affect de novo or salvage dNTP synthesis, or hydrolysis. Genetic syndromes have been described that interfere with RNR function. RNR is composed of large (R1) and small (R2) subunits but in mammalian cells there is an additional specialised R2 subunit, p53R2. This subunit is expressed at a lower level than the normal R2 subunit and also differs in that it is not degraded in mitosis, and thus its level is constant during the cell cycle and may provide dNTPs for repair [108]. However, mutations in the *RR2MB* gene encoding p53R2 cause mtDNA deficiency syndromes [101,109,110], indicating that an important function of R1/p53R2 is the provision of basal levels of deoxynucleotides outside of S phase which are imported from the cytoplasm into mitochondria to support mtDNA replication.

Nucleotide salvage pathways are also important for maintenance of mtDNA and defects in dNTP salvage enzymes thymidine kinase 2 and deoxyguanosine kinase lead to mtDNA depletion syndromes [111,112], reviewed in [113]. An additional mtDNA depletion syndrome, MNGIE, is caused by mutations affecting function of the cytoplasmic enzyme thymidine phosphorylase (TP). This leads to an elevated level of dTTP in mitochondria, which is not deleterious in itself but leads to secondary dCTP depletion and inhibition of mtDNA replication [114]. Mutations in mtDNA replication enzymes can also lead to dNTP level changes; mutations affecting the TWINKLE helicase derange cellular dNTP levels, contributing to mtDNA mutagenesis [115]. Nuclear genome instability can also be affected by mutations affecting salvage enzymes. BLM helicase deficiency is associated with cytidine deaminase downregulation, pyrimidine pool disequilibrium, and reduced fork rate [116]. Loss of Fhit expression, owing to FRA3B fragile site deletions, leads to downregulation of thymidine kinase I expression and increased genetic instability [117].

Mutations affecting dNTP levels may also contribute to genetic instability and cancer development. Overexpression of the R2 subunit of RNR (RRM2 or p53R2) but not the R1 subunit in mice leads to an increase in the mutation frequency and lung neoplasms [118]. This may reflect the fact that the R2 subunit is regulated by proteolysis, and its overexpression results in higher dNTP levels and increased mutagenesis [119]. It has been additionally suggested that this phenotype may be due to the ability of the R2 subunit to generate a tyrosyl radical that might lead to reactive oxygen species (ROS) and oxidative DNA damage (reviewed in [95]). High levels of dNTPs can also arise through defects in SAMHD1 and may lead to cancer development (reviewed in [120]). Colon cancer is associated with mutations in MMR [121,122] and, less frequently, with proofreading mutations in replicative polymerases [123], but an additional link has been reported with SAMHD1 [124]. Mutations that inactivate just one allele of SAMHD1 can significantly increase dNTP pools and may exacerbate the phenotype of MMR mutations [124]. Mutations in SAMHD1 also cause the autoimmune Aicardi–Goutières syndrome [125], possibly related to the nuclease activity of the enzyme [126], allowing accumulation of interferon-stimulating ssDNAs in SAMHD1-deficient cells. dCTP hydrolyzing enzymes have also been described that may have a role in regulating dNTP pools, or sanitizing dCTP pools by eliminating modified nucleotides, and it will be interesting to determine if defects in these enzymes have a role in tumour progression or other genome stability defects [127,128].

In addition to mutations affecting dNTP supply directly, genomic instability may result from oncogenic mutations that upregulate proliferation without compensatory changes that also increase nucleotide supply, and such instability may further contribute to evolution of cancer cells. Mutations in proto-oncogenes may cause S phase stress via stalling and collapse of replication forks [129], leading potentially to senescence or apoptosis via p53 activation. The high frequency of p53 inactivation found in tumours could allow such oncogene-induced stress to contribute to ongoing genomic instability necessary for tumour development. More insight into the cause of oncogene-induced replication stress comes from the observation that induction of the Rb-E2F pathway causes dNTP depletion in transformed cells and, intriguingly, exogenously supplied nucleosides can rescue the replication stress and reduce transformation [130]. Evidence for S phase problems in this situation additionally comes from poor processivity of the replication fork, and there may be enhanced defects at hard-to-replicate sites [130].

Cell-type differences may also be an important physiological factor in dNTP-mediated replication stress. During development, rates of cell proliferation or S phase duration may vary considerably between different progenitor cell types and as a result some lineages may be exposed to more replication stress. Nervous system development appears to be acutely sensitive to defects in DNA repair, in part reflecting replication-associated DNA damage (reviewed in [131,132]), and it is possible that dNTP-imposed replication stress is a significant factor in neurogenic lineages. Cell-type differences in metabolic pathways that feed into dNTP synthesis may also be a factor in replication stress. For instance, fatty acid oxidation is important for dNTP synthesis in endothelial cells, and blocking fatty acid oxidation may thus present a therapeutic strategy for impairing pathogenic angiogenesis [133].

At the cellular level, local reductions in nuclear dNTPs may not be adequately balanced by diffusion of nucleotides from the cytoplasm, where active RNR is situated, leading to replication stress. Replication of simple sequences, such as repeats of complementary nucleotides, may cause local starvation of specific dNTPs [134]. Thus consumption of, for instance, dATP and dTTP from replicating AT-rich DNA may lead to local imbalance of dNTP levels, resulting in polymerase stalling, potentially leading to microsatellite instability [135].

## 7. Future Directions

**Figure 2:**
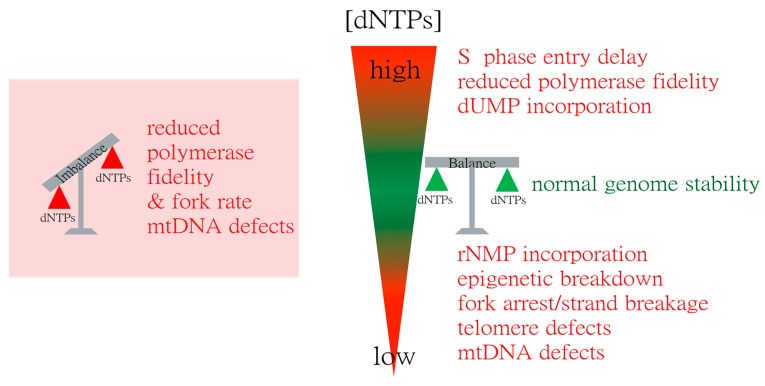
Summary of consequences of abnormal dNTP levels.

It is clear that maintenance of physiological dNTP levels is critical for genome stability and the consequences of altered nucleotide levels are complex (summarised in Figure 2). These can be investigated with precision in model organisms such as yeasts, and the mechanistic insights gained can be tested in mammalian cells to determine evolutionary conservation. One controversial issue that has not been resolved by current methodologies is the relevance of the site of dNTP synthesis to the sites of nuclear dNTP consumption. A wide body of evidence indicates that dNTP synthesis by RNR is cytoplasmic and it is assumed that rates of dNTP diffusion are sufficient to provide for nuclear DNA synthesis ([136] and references cited within). However, a number of recent findings indicate that at least for DNA repair, RNR is targeted to sites of DNA damage [54,108,119,137,138]. One interpretation of these findings is that dNTP diffusion may be potentially limiting for DNA synthesis and under some circumstances it may be advantageous to synthesize dNTPs at the site where they are needed. However, it is difficult to critically assess this issue due to limitations in accurately determining dNTP concentrations. Currently measurements are made on cell populations, and even with synchronized cells this may not give an accurate indication of levels of dNTPs available to polymerases (e.g., see reference [68]). Measurements of dNTP levels in single cells would clarify the degree of cell-to-cell variation in nucleotide homeostasis. Fluorescence methods could allow monitoring of how subcellular variations in dNTP levels are affected by nuclearly-targeted RNR, and other factors such as replication of simple repeat sequences [134,135], high density of replication forks, and nuclear positioning of chromosomal segments. Micro-inhomogeneities in dNTP levels could be another factor promoting genomic instability in the absence of global perturbations in nucleotide supply.
